# One-Pot Hydrothermal Synthesis of Magnetite Prussian Blue Nano-Composites and Their Application to Fabricate Glucose Biosensor

**DOI:** 10.3390/s16020243

**Published:** 2016-02-18

**Authors:** Ezzaldeen Younes Jomma, Shou-Nian Ding

**Affiliations:** 1 School of Chemistry and Chemical Engineering, Southeast University, Nanjing 211189, China; ezzoezzo11@yahoo.com; 2 Food Technology Department, Nyala Technological College, Nyala P. O. Box 155, Sudan

**Keywords:** magnetite Prussian blue nano-composites, one-pot synthesis, biosensor

## Abstract

In this work, we presented a simple method to synthesize magnetite Prussian blue nano-composites (Fe_3_O_4_-PB) through one-pot hydrothermal process. Subsequently, the obtained nano-composites were used to fabricate a facile and effective glucose biosensor. The obtained nanoparticles were characterized using transmission electron microscopy, scanning electron microscopy, Fourier-transform infrared spectroscopy, UV-vis absorbance spectroscopy, cyclic voltammetry and chronoamperometry. The resultant Fe_3_O_4_-PB nanocomposites have magnetic properties which could easily controlled by an external magnetic field and the electro-catalysis of hydrogen peroxide. Thus, a glucose biosensor based on Fe_3_O_4_-PB was successfully fabricated. The biosensor showed super-electrochemical properties toward glucose detection exhibiting fast response time within 3 to 4 s, low detection limit of 0.5 µM and wide linear range from 5 µM to 1.2 mM with sensitivity of 32 µA∙mM^−1^∙cm^−2^ and good long-term stability.

## 1. Introduction

Since Clark and Lyons reported their first enzyme electrode for glucose determination in 1962, a tremendous attention has been given to develop electrochemical biosensors via immobilization of the enzymes on an electrode surface [1,2].

Among the electrochemical enzyme biosensors, the glucose biosensors have been developed as commercial clinical devices to monitor the glucose concentration of real time in human being blood. In the mechanism of glucose biosensor, the oxidation of glucose can be catalyzed by glucose oxidase (GOD) in the presence of oxygen to produce gluconic acid and hydrogen peroxide (H_2_O_2_) in which the generated H_2_O_2_ can be detected by electrochemical method. However, the detection of H_2_O_2_ at high potential suffers from the oxidation of interferes such as dopamine, uric acid, and ascorbic acid.

Prussian blue (PB) the simplest representative of MHCF family and its different analogues have been utilized worldwide for fabrication of chemical sensors/biosensor as transducers for the reduction of hydrogen peroxide at low applied potential [3,4]. For a glucose biosensor, the generated H_2_O_2_ can be detected by PB which exhibits high activity and selectivity [5]. Despite the attractive properties of PB, the PB films are unstable over long time under neutral or alkaline conditions, which greatly limits their applications in non-acidic solutions [6,7].

The advance in magnetic nanoparticles is their easy separation and purification by an external magnet [8]. Several methods have been used to synthesize magnetic nanoparticles, such as co-precipitation, hydrothermal synthesis, thermal decomposition and/or reduction, sol-gel reaction, electrochemical methods, flow injection synthesis and aerosol/vapor [9,10]. Among them, hydrothermal method has the ability to create crystalline phases which are not stable at the melting point. In addition, materials which have a high vapor pressure near their melting points can also be grown by hydrothermal method. Moreover, the hydrothermal method is also particularly suitable for the growth of high-quality nanoparticles while maintaining good control over their composition [11].

Many procedures of synthesis magnetic PB nanoparticles have been reported in the literature. Ge *et al.* prepared PB-Fe_3_O_4_ nanoparticles in two step procedure, first synthesized of Fe_3_O_4_ by co-precipitation of Fe^2+^ and Fe^3+^ ions in the presence of NaOH at 80 °C then the obtained Fe_3_O_4_ particles were suspended in K_3_Fe(CN)_6_ + FeCl_3_ + HCl solution under stirring to form PB-Fe_3_O_4_ [12]. Recently, Ge *et al.* synthesized Fe_3_O_4_@PB NPs by improving shell growing procedure by mixing aqueous dispersion of Fe_3_O_4_ with aqueous solution of K_4_Fe(CN)_6_ in acidic condition under mechanical stirring at room temperature. Subsequently, the aqueous solution of FeCl_3_ (pH 3) was added into above mixed to form Fe_3_O_4_@PB NPs [13]. Li *et al.* were also synthesized Fe_3_O_4_ NPs containing PB NPs in which Fe_3_O_4_ nanoparticles were prepared by co-precipitating of Fe^2+^ and Fe^3+^ ions then the obtained Fe_3_O_4_ particles were re-dispersed by sonication in K_3_Fe(CN)_6_ solution (pH 2) followed by addition of H_2_O_2_ as reducing agent and the mixture was stirred for 3 h to form Fe_3_O_4_/PB complex [2]. Zhang *et al.* presented a simple approach to synthesize γ-Fe_2_O_3_ modified PB at different levels. The γ-Fe_2_O_3_ NPs were first prepared then the obtained particles were diluted in deionized water (pH 2) and used as a seeds to prepare γ-Fe_2_O_3_-PB NPs. An aqueous solution of K_4_Fe(CN)_6_ with different volumes was added dropwise to the γ-Fe_2_O_3_ solution under stirring for 1 h [14].

In the past decades, the synthesis of magnetic-PB NPs has been developed not only for its fundamental scientific interest but also for many technological applications. They has been used in bio-sensing application such as glucose biosensor [2], targeted photothermal therapy of cancer under magnetic resonance imaging (MRI) [13], H_2_O_2_ sensor [12,15] and the removal of cesium from contaminated environment [8]. Recently, the strategies focus on developing these materials in the sensors fabrication because PB, a prototype of mixed valence transition metal hexacyanoferrate is an ancient dye with low cost, good catalytic activities and simple preparation. Moreover, the good characteristic of the reduced form of PB, Prussian White, to catalyze the reduction of H_2_O_2_ at low applied potential can minimize the effects of the most common electrochemical interfering species, which leads interest of research to develop sensors and biosensors based on PB nanocomposite materials. The magnetic nanoparticles have also exhibited substantial application in biomedicine as an enhancement agent especially for the magnetic resonance imaging. The most prominent and the decent characteristics of these nanoparticles are the ability to form stable colloidal suspensions in water without any organic or inorganic coating to prevent their aggregation and, at the same time, they are able to bind specific organic molecules leading to composite materials, which can be exploited for biotechnological applications. Furthermore, nanocomposite magnetic PB materials showed remarkable electrocatalytic properties and were used for the development of sensor and biosensor for hydrogen peroxide and glucose detection with high sensitivity, low detection limit, good reproducibility and stability.

Regardless the interesting properties of magnetic nanoparticles, the applications of pure magnetic nanoparticles are limited due to agglomeration of the fine particles for their large ratio of surface area to volume. The pure magnetic particles are also easily oxidized in air, therefore, losing the magnetic properties [9,12]. The modification of Fe_3_O_4_ particles with polymers, metals and semiconductor materials has been widely involved to overcome these problems.

The composite nanomaterials formed from magnetic Fe_3_O_4_ nanoparticles and PB would be promising to overcome such problems and be interesting to fabricate glucose biosensors for clinical applications in the human body due to the reliable biosafety [13].

In this work we synthesized magnetite PB nano-composites in one pot hydrothermal procedure. Subsequently, we introduced the magnetite PB nano-composites to fabricate electrochemical glucose biosensor via immobilization of GOD-BSA by cross-linking with glutaraldehyde vapor on Fe_3_O_4_-PB modified glassy carbon electrode (GCE) surface denoted as (GOD-BSA/Fe_3_O_4_-PB/GCE). The combination of Fe_3_O_4_-PB nanocomposite and enzyme significantly enhanced the performance such as stability and sensitivity in enzyme biosensor.

## 2. Experimental

### 2.1. Chemicals and Reagents

Glucose oxidase (GOD, EC 1.1.3.4 purified from *Aspergillus Niger*) was purchased from Sigma (Shanghai, China). Bovine serum albumin (BSA) was bought from Sangon Chemical Company (Shanghai, China). Glutaraldehyde (GA), potassium ferricyanide and potassium chloride were obtained from Shanghai Lingfeng Chemical Company (Shanghai, China). D-(+)-glucose, Iron (III) chloride hexahydrate, hydrogen peroxide and ethylene glycol were obtained from Sinopharm Chemical Company (Shanghai, China) and polyethylene glycol 4000 was obtained from Shantou Xilong Chemical Co., Ltd (Shantou, China).

### 2.2. Instruments

The magnetite PB particles were examined by transmission electron microscope (Gatan, Inc., Pleasanton, CA, USA) and scanning electron microscopy (SEM) and energy dispersive X-ray spectroscopy (EDAX) images were taken with scanning electron microscope (S-3000 N, Hitachi High-Technologies Corporation, Tokyo, Japan). Fourier-transform infrared (Nicolet 5700 FTIR spectrometer, Thermo elctron corporation, Madison, WI, USA ) and UV-Vis absorbance spectroscopy using UV-2450 spectrophotometer (Shimadzu Corporation, Kyoto, Japan) were performed to characterize the magnetite PB particles. Cyclic voltammetry and chronoamperometry were carried out on electrochemical workstation (CHI821C, Shanghai Chenhua Co., Ltd., Shanghai, China). The modified glassy carbon electrode (3.0 mm in diameter) was used as the working electrode, a saturated calomel electrode (SCE) as the reference electrode and a platinum wire as the auxiliary electrode.

### 2.3. One-Pot Synthesis of Magnetite PB Nanoparticles

**Scheme 1:**
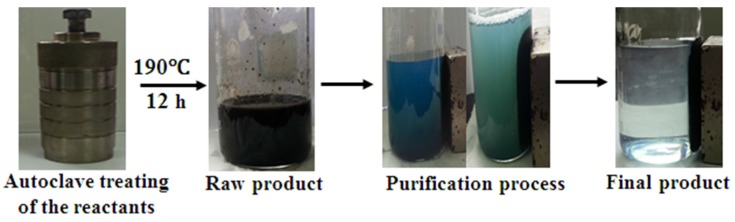
Synthesis and purification process of the magnetite Prussian blue (PB) nano-composites.

All of the reagents in this experiment are analytical grade and used without further purification. Magnetite PB nanoparticles were synthesized via one-pot hydrothermal reduction of FeCl_3_·6H_2_O and K_3_Fe(CN)_6_ by the ethylene glycol (EG) in the presence of polyethylene glycol (PEG) as surfactant. Typically, FeCl_3_·6H_2_O 1.3 g (0.124 M) was dissolved in 40 mL of EG followed by addition of 1.0 g PEG under mechanical stirring. 0.67 g K_3_Fe(CN)_6_ (0.049 M) was added to the above solution and continued stirred vigorously for 1 h and then transferred into Teflon-lined stainless-steel autoclave. The autoclave was then heated and maintained at 190 °C for 12 h. Afterward the autoclave was allowed to cool overnight at room temperature. The precipitation was collected by magnetic separation, washed several times with distilled water and dried for 6 h in oven at 60 °C. The preparation and purification process has been illustrated in Scheme 1.

### 2.4. Preparation of Glucose Oxidase Biosensor on Magnetite PB Modified Glassy Carbon Electrode

**Scheme 2:**
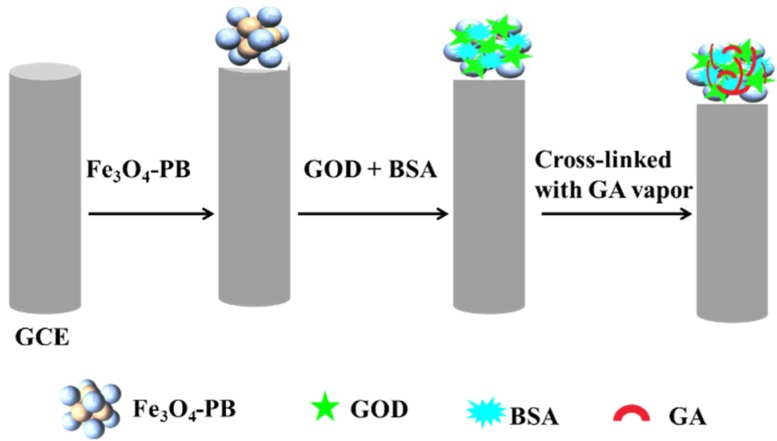
Steps to fabricate the biosensor.

The GCE was polished before each experiment with 1.0, 0.3, and 0.05 µm α-alumina powder, respectively, rinsed thoroughly with double distilled water. 1 mg/mL of synthesized magnetite PB nanocomposite was dispersed in double distilled water and then 6 µL of magnetite PB solution was dropped onto the cleaned GCE surface allowed to dry at room temperature followed by heating for 1 h at 100 °C denoted as (Fe_3_O_4_-PB/GCE). GOD (5 mg/mL) was prepared in PBS (pH 6.5); BSA (5 mg/mL) was also prepared in PBS (pH 6.5). Enzyme mixture was prepared by mixing (1:1 V/V) GOD and BSA then 5 µL of enzyme mixture was dropped onto the obtained Fe_3_O_4_-PB/GCE and allowed to dry at 4 °C in refrigerator then the electrode was introduced to glutaraldehyde (25%) vapor for cross-linking of enzyme mixture for 15 min. The final modified electrode is denoted as GOD-BSA/Fe_3_O_4_-PB/GCE. The biosensor preparation steps have been shown in Scheme 2.

## 3. Results and Discussion

### 3.1. Possible Formation Mechanism of Magnetite PB Nanocomposite

We prepared magnetite PB nanoparticles by one-pot hydrothermal route using ferric (III) chloride and potassium ferricyanide as precursors, ethylene glycol as solvent and reducing reagent and PEG as protective reagent to keep nanoparticles from agglomeration. During the hydrothermal process, EG can reduce some of Fe^3+^ ions to Fe^2+^ ions to form Fe(OH)_3_ and Fe(OH)_2_ by hydrolysis firstly. The hydroxides finally become Fe_3_O_4_ nanoparticles by thermal decomposition [16,17]. Concerning to the formation of magnetite PB, there are two possibilities: (1) The above-formed Fe^2+^ ions in the reaction medium could react with Fe(CN)_6_^3−^ to form PB nanoparticles, which precipitate onto Fe_3_O_4_ nanoparticles surface to form the final nanocomposites via the electrostatic attraction between Fe_3_O_4_ nanoparticles and PB nanoparticles [18]; (2) Fe(CN)_6_^3−^ might be reduced to Fe(CN)_6_^4−^, which can react with Fe^3+^ ions adsorbed on the formed Fe_3_O_4_ nanoparticles surface to form the final products. After the hydrothermal reaction, the color of the product was dark blue and the formed particles could be separated by external magnet indicating the magnetic PB nanocomposites have been successfully prepared (Scheme 1).

### 3.2. Characterization

**Figure 1:**
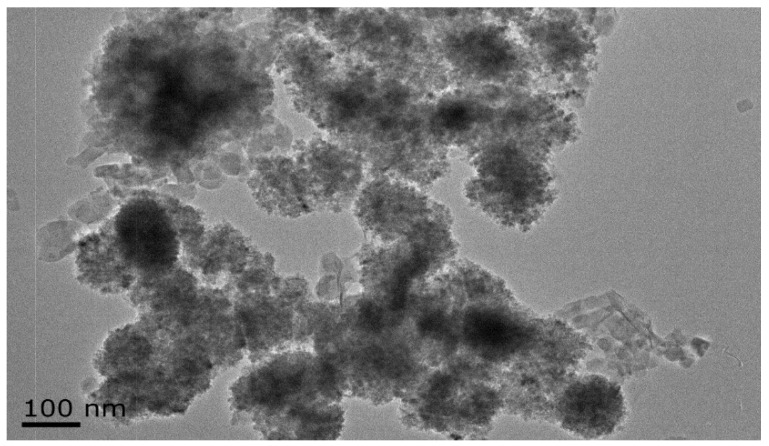
TEM image of the magnetite PB nano-composites.

**Figure 2:**
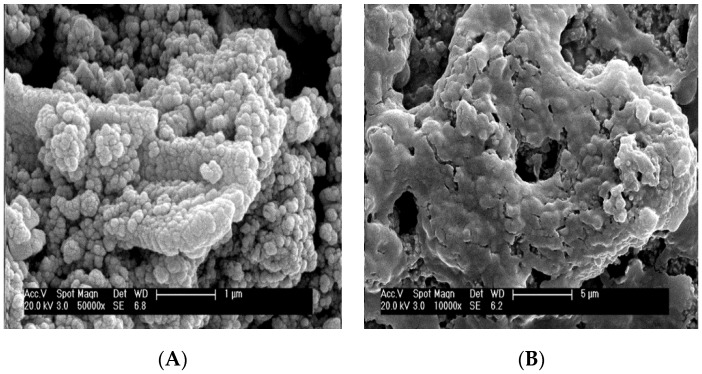
SEM images of (**A**) Fe_3_O_4_-PB film and (**B**) Glucose oxidase (GOD)-bovine serum albumin (BSA)/Fe_3_O_4_-PB film.

The morphologies of the magnetite PB nanocomposites were characterized by TEM. Figure 1 shows the TEM results of the typical synthesized magnetite PB nanocomposites. Numerous spherical and cubic nanoparticles combined compactly together were observed. The spherical and cubic nanoparticles belong to Fe_3_O_4_ and PB, respectively. Meanwhile, Fe_3_O_4_-PB nanocomposites thin film and GOD-BSA/Fe_3_O_4_-PB thin film modified electrodes were characterized by SEM. Figure 2 shows their SEM images. As seen in Figure 2A, the morphologies of Fe_3_O_4_-PB nanocomposites were consistent with the results from their TEM measurements. After the Fe_3_O_4_-PB modified electrode was coated with GOD and BSA mixture via cross-linking by glutaraldehyde, it is apparent to observe an enzyme membrane covered on Fe_3_O_4_-PB film (Figure 2B). To further verify the formation of magnetite PB nano-composites, EDAX was performed upon magnetite PB nanocomposites modified platinum sheet. As seen in Figure S1, (in the supplementary materials), elements content of the nanocomposites showed the existing of C, N, O and Fe, which are typical elements of Fe_3_O_4_ and PB.

**Figure 3:**
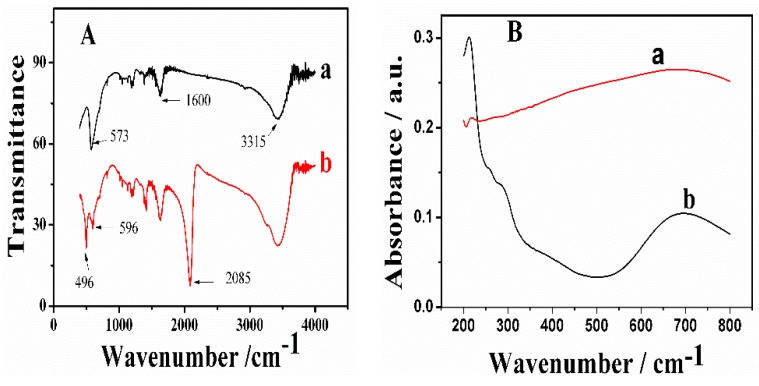
(**A**) FTIR spectra of pure Fe_3_O_4_ nanoparticles (**curve a**) and magnetite PB nano-composites (**curve b**); (**B**) UV-vis absorption spectra of Fe_3_O_4_ nanoparticles (**curve a**) and magnetite PB nano-composites (**curve b**).

As shown in Figure 3A, FTIR was also used to characterize Fe_3_O_4_ (Figure 3A, curve a) and magnetite PB nanoparticles (Figure 3A, curve b). In Figure 3A, curve a, the absorption band at 573 cm^−1^ is assigned to the absorption peak of Fe–O bonds [19]. The absorption peaks at around 3315 and 1600 cm^−1^ are related to O–H and H–O–H, respectively, which are attributed to the existence of interstitial water in the particles [12]. For the magnetite PB particles (Figure 3A, curve b), the absorption band at 2085 cm^−1^ are attributed to the CN group, the absorption band at 496 cm^−1^ belongs to the Fe–CN–Fe [8,12,20], and the absorption peak at 596 cm^−1^ refers to the Fe–O bond. Existence of peaks at 2085, 496, and 596 cm^−1^ confirmed the formation of magnetite PB nanoparticles through one pot hydrothermal synthesis. The Fe–O band shifted from Fe_3_O_4_ to magnetite PB to higher wavenumbers of 596 cm^−1^. This phenomenon is indicating the formation of the nanocomposite. One of the basic influences of the finite size of NPs is the breaking of a large number of bonds for surface atoms, resulting in the rearrangement of dissociative electrons on the particle surface. Hence, when particles are reduced to nanoscale dimensions, the absorption bands of FTIR spectra shift to higher wavenumbers [14].

UV-vis spectroscopy was further used to investigate the formation of the magnetite PB composite as shown in Figure 3B. For pure Fe_3_O_4_ nanoparticles (Figure 3B, curve a), no clear absorbance peaks could be observed due to its wide spectrum absorbance property. However, for the magnetite PB composite (Figure 3B, curve b), the mixed valence charge transfer band of the PB Fe^II^–C–N–Fe^III^ at around 740 nm was observed. Both results obtained from FTIR and UV-vis showed good agreement to the results reported in the literature [21,22].

### 3.3. Electrochemical Behaviors of Fe_3_O_4_-PB and GOD-BSA/Fe_3_O_4_-PB/GCE

**Figure 4:**
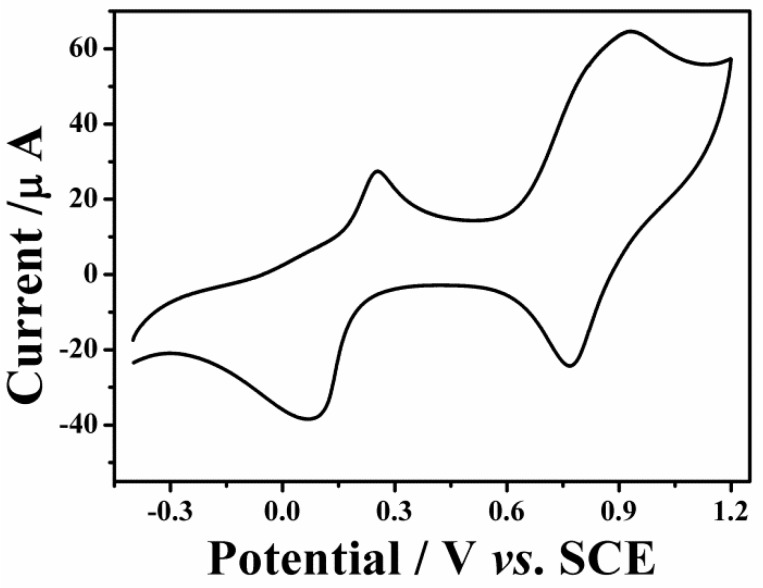
Cyclic voltammogram (CV) of the magnetite PB nano-composites modified glassy carbon electrode (GCE) in 0.1 M PBS (pH 6.0) with the scan rate of 100 mV∙s^−1^.

To investigate the electrochemical behaviors of the synthesized magnetite PB nanocomposites and its corresponding glucose oxidase biosensor, cyclic voltammetry and chronoamperometry were performed. As shown in Figure 4, two pairs of anodic and cathodic peaks were observed from cyclic voltammogram (CV) of Fe_3_O_4_-PB/GCE in 0.01 M PBS (pH 6.0) containing 0.1 M KCl at the scan rate of 100 m∙Vs^−1^. The two pair of peaks were attributed to the oxidation of PB to Prussian green (PG) and the reduction of PB to Prussian white (PW), respectively. Clearly, from the shape of CV, the synthesized nano-composites contain PB [23].This result is with good agreement to the above-mentioned other characterization.

The conventional PB modified electrodes suffer from the instability of the film in neutral and alkaline conditions due to the presence of hydroxide ions, which could break Fe–(CN)–Fe bonds. Therefore, we investigated the pH effect to magnetite PB modified electrode in PBS with the pH values ranging from 5 to 9. Figure S2 (supplementary materials) showed multi-CVs of Fe_3_O_4_-PB/GCE in different PBS at scan rate of 100 mV∙s^−1^. The modified electrodes exhibited good stabilities in pH 5 to 7. However, the current peaks showed a little decrease in pH 8 and 9 upon 20 potential scanning cycles on each pH value. The current peak is almost unchanged which reveals that no proton is involved in the electrochemical reaction of PB. As well-known, the unmodified PB film is highly unstable in neutral and alkaline solution, and it has significant loss of redox activity. The good stability of the Fe_3_O_4_-PB/GCE may be due to the presence of the magnetic ions on the magnetite PB nano-composites which block the attack of hydroxide ions towards PB. In conclusion, electrostatic interaction between Fe_3_O_4_ and PB may offer stabilization mechanism to prevent its dissolution.

**Figure 5:**
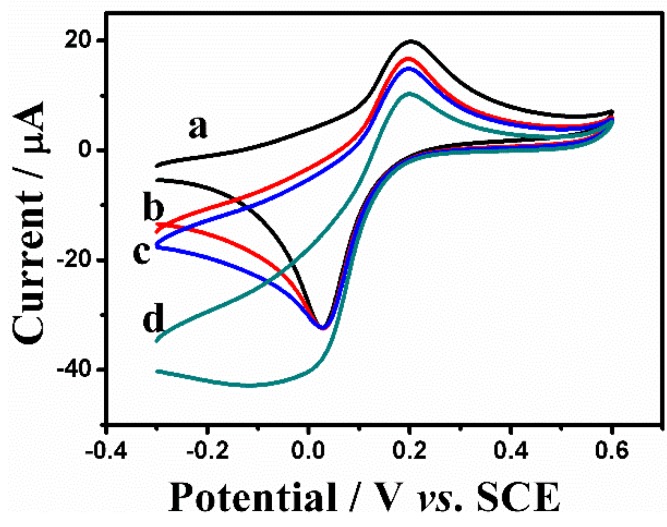
CVs of magnetite PB nano-composites modified GCE in the absence (**a**) and presence of 1 mM (**b**), 2 mM (**c**), and 4 mM H_2_O_2_ (**d**) in 0.1 M PBS (pH 6.0) with the scan rate of 100 mV∙s^−1^.

The electro-catalysis of Fe_3_O_4_-PB/GCE to H_2_O_2_ was investigated by cyclic voltammetry. Figure 5 shows the CVs of the Fe_3_O_4_-PB/GCE upon different concentrations of H_2_O_2_ in 0.01 M PBS (pH 6.0) containing 0.1 M KCl. The potential scan range was between –0.3 and 0.6 V *vs.* SCE and the scan rate is 100 mV∙s^−1^. The reduction current peaks increased significantly in magnitude upon increasing concentrations of H_2_O_2_ and the oxidation peaks decreased correspondingly, which demonstrated the electrocatalytic reduction of H_2_O_2_.

As shown in Figure S3 (in the supplementary materials), the amperometric determination of H_2_O_2_ was performed by successive addition of H_2_O_2_ into 0.01 M PBS (pH 6.0) containing 0.1 M KCl under stirring at –0.1 V. The amperogram of the Fe_3_O_4_-PB/GCE displayed fast response and stable signal with injection of H_2_O_2_. The response time is only 4–5 s.

Compared with Fe_3_O_4_-PB/GCE, an obvious decrease of peak currents was observed in the GOD-BSA/Fe_3_O_4_-PB/GCE as shown in Figure S4 (in the supplementary materials). This was attributed to the block effect of enzyme membrane to the mass transportation, which is consistent with the SEM measurement (Figure 2).

**Figure 6:**
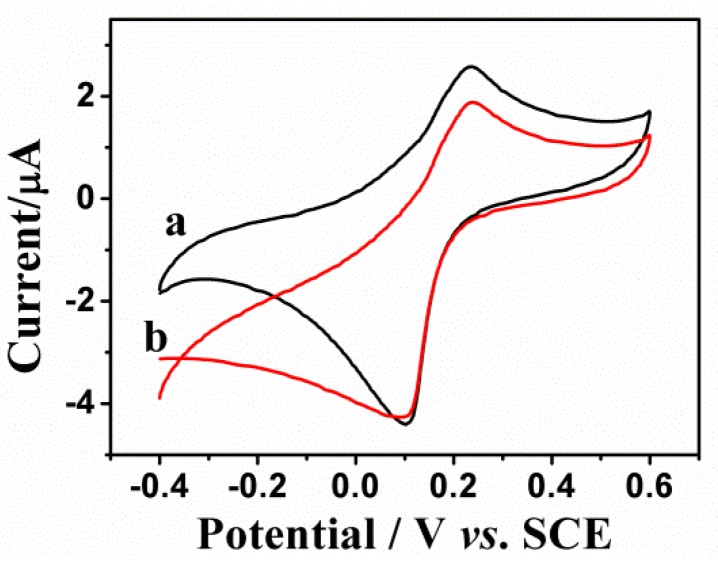
CVs obtained at the GOD–BSA/Fe_3_O_4_-PB/GCE in 0.01 M PBS (pH 6.0) containing 0.1 M KCl in the absence (**a**) and presence (**b**) of 1 mM glucose with the scan rate of 50 mV s^−1^.

Figure 6 showed the typical CVs of GOD-BSA/Fe_3_O_4_-PB/GCE for glucose detection. In the presence of 1 mM glucose, the cathodic peak current increased and the anodic peak current decreased indicating catalytic reduction reaction. The description for this reaction process is that glucose diffuses from the electrolyte solution to the enzyme film on the electrode surface then GOD catalyzes the oxidation of glucose resulting in the generation of H_2_O_2_ and gluconic acid in consumption of oxygen. Subsequently, the generated H_2_O_2_ is then catalytically reduced by Prussian white (the reduced form of PB) [24].

### 3.4. Detection of Glucose

For the fabrication of GOD-BSA/Fe_3_O_4_-PB/GCE, the effect of cross-linking time in glutaraldehyde vapor was investigated from 5 min to 30 min. The optimal time is 15 min. A short time in the vapor leads the instability of the enzyme membrane due to the insufficient cross-linking. A long time in glutaraldehyde causes the decreasing of the bioactivity of GOD. For the detection of glucose, the applied potential of –0.15 V was chosen to perform the current-time response experiment due to the acceptable sensitivity and avoiding the interference of electroactive species shown in Figure S5. The sensitivity and the bioactivity of the biosensor greatly depend on the solution pH values. The current-time response of GOD-BSA/Fe_3_O_4_-PB/GCE in PBS with different pH values (from 4 to 9) with the injection of 0.4 mM glucose was investigated. The current peak response reached its maximum at pH 6.0, which is consistent with the optimal enzyme activity and the performance of Fe_3_O_4_-PB in acidic condition. Hence, the pH 6.0 is selected as the optimal value for this biosensor.

**Figure 7:**
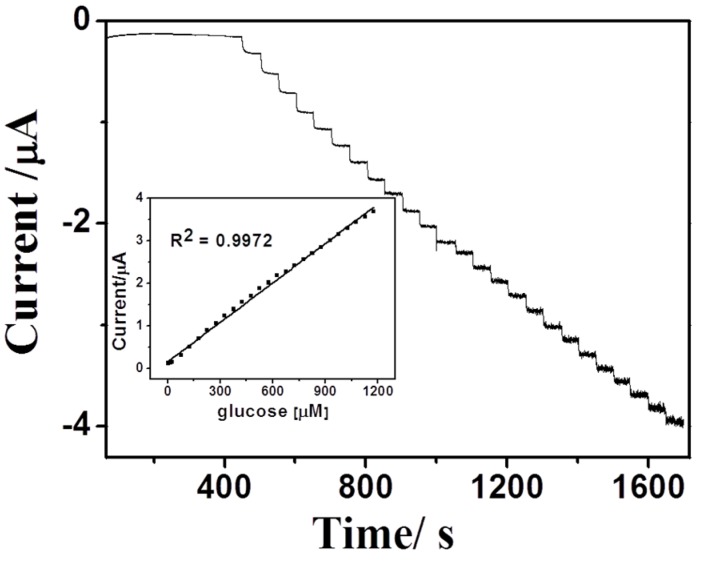
Current-time response of the biosensor upon successive addition of different concentrations of glucose from 5 µM to 1500 µM in 0.01 M PBS (pH 6.0) containing 0.1 M KCl under stirring. The applied potential was −0.15 V. Inset: the catalytic response *vs.* glucose concentration.

Under the optimal conditions, the current-time response of GOD-BSA/Fe_3_O_4_-PB/GCE upon successive addition of glucose (0.5 µM and 1500 µM) in 0.01 M PBS (pH6.0) containing 0.1 M KCl under stirring was shown in Figure 7. The electrocatalysis current reached 95% of its steady current within short time about 3–4 s after the injection of glucose indicating the biosensor has fast response. Meanwhile, the biosensor exhibited wide linear range to glucose concentration from 5 to 1200 µM with a sensitivity of 32 µA∙mM^−1^∙cm^−2^ and the limit of detection of 0.5 µM. The inset in Figure 7 displayed the corresponding calibration curve for glucose. The wide linear range of this biosensor may be due to the big loading of enzymes using the cross-linking method. The usage of BSA might not only provide sufficient amino groups for cross-linking via glutaraldehyde to stabilize GOD but also provide biocompatible surroundings to maintain the bioactivity of GOD. Table 1 shows the performance comparison of this work with other recently reported biosensors in the term of detection limit, linear range, sensitivity and response time.

### 3.5. Storage Stability of the Biosensor

In order to investigate the storage stability of the biosensor, cyclic voltammetry was used to detect constant concentration of glucose daily for two weeks. The biosensor showed acceptable stability with decreasing in current peaks about 5%–8% from its initial value. The long term stability of the biosensor attributed to the stability of the Fe_3_O_4_-PB nanocomposite film and the cross-linking enzyme membrane allowed large loading of the enzyme.

## 4. Conclusions

Electroactive Fe_3_O_4_-PB nano-composites have been synthesized in one-pot hydrothermal approach for the first time. Fe_3_O_4_-PB nano-composites modified GCE exhibited good performance in electro-catalysis of hydrogen peroxide, especially its stability. Furthermore, a highly sensitive glucose biosensor was developed via cross-linking of GOD-BSA onto Fe_3_O_4_-PB nano-composites modified GCE. The cross-linking between BSA and GOD guaranteed for more loading and good stability of GOD. The association of magnetite PB could improve the stability of PB film. Obviously, The Fe_3_O_4_-PB nano-composites film could be used as a promising platform for the development of biosensors related to hydrogen peroxide.

## Figures and Tables

**Table 1:** Comparison of electrocatalytic activities of this work with the previous reported.

Working Electrode	LOD (µM)	Linear Range	Sensitivity	Response Time (s)	Reference
PB/GOD/TEOS-PVA/NAFION	20	5–1000 µM	2.06 µA∙mM^−1^∙cm^−2^	No data	[25]
Anodisc™/Pt/PPy/GOx nanotube arrays	50	0.2–13 mM	72.1 mA∙M^−1^∙cm^−2^	13	[26]
Fe_3_O_4_/PB/GOD	0.1	0.5–80 µM	No data	10	[2]
GOx/Chi-IL/PB/Pt	5	0.01–4.2 mM	37.8 µA∙mM^−1^∙cm^−2^	3	[27]
GOx–PDA/PB/GCE	46.2	0.2–3.4 mM	1.59 nA∙µM^−1^	15	[28]
PBNPs-PANI/MWNTs/GCE	0.6	6.7–1900 µM	6.28 µAmM^−1^	5	[29]
PVA-Au-pphTEOS-GOD	700	1–8 mM	49 µA∙mM^−1^∙cm^−2^	6	[30]
Alg/LDHs/GOD/Pt	4	0.016–2 mM	68.9 mA∙M^−1^∙cm^−2^	10	[31]
GOD-BSA/GA/Fe_3_O_4_-PB/GCE	0.5	5–1200 µM	32 µA∙mM^−1^∙cm^−2^	4	This work

## Supplementary Materials

The following are available online at http://www.mdpi.com/1424-8220/16/2/243, Figure S1: EDAX showing the elements content of the composite Fe_3_O_4_-Prussian blue. Figure S2: Multi-CVs of the Fe_3_O_4_-PB/GCE in 0.01 M Phosphate buffer solution (PBS) containing 0.1 M KCl at deferent pH values (**A**) 5.0, (**B**) 6.0, (**C**) 7.0, (**D**) 8.0, (**E**) 9.0. Figure S3: Current-time response of the Fe_3_O_4_-PB/GCE to the successive addition of H_2_O_2_ in 0.01 M PBS (pH 6.0) + 0.1 M KCl under stirring at −0.1 V. Insert: Plot of catalytic current *vs.* H_2_O_2_ concentration. Figure S4: Typical CVs obtained at Fe_3_O_4_-PB/GCE (**A**) and GOD-BSA/Fe_3_O_4_-PB/GCE (**B**) in 0.01 M PBS (pH 6.0) + 0.1 M KCl. Scan rate: 50 mV∙s^−1^. Figure S5: Typical Chronoaperometry (I-t) response of 0.025 µM glucose and (**A**) 0.1 µM AA and 0.1 µM UA (**B**) 0.2 µM AA and 0.2 µM UA at GOD-BSA/Fe_3_O_4_-PB/GCE. Applied Potential: −0.15 V.
