# Supplementary material for: One-Pot Hydrothermal Synthesis of Magnetite Prussian Blue Nano-Composites and Their Application to Fabricate Glucose Biosensor
Source: Sensors (Basel). 2016 Feb 18;16(2):243. doi: 10.3390/s16020243 (PMC4801619; doi:10.3390/s16020243)
Supplement: Supplementary file 1 [file sensors-16-00243-s001.pdf]

Supplementary Materials: One-Pot Hydrothermal Synthesis of Magnetite Prussian Blue Nano-Composites and Their Application to Fabricate Glucose Biosensor

Ezzaldeen Younes Jomma and Shou-Nian Ding

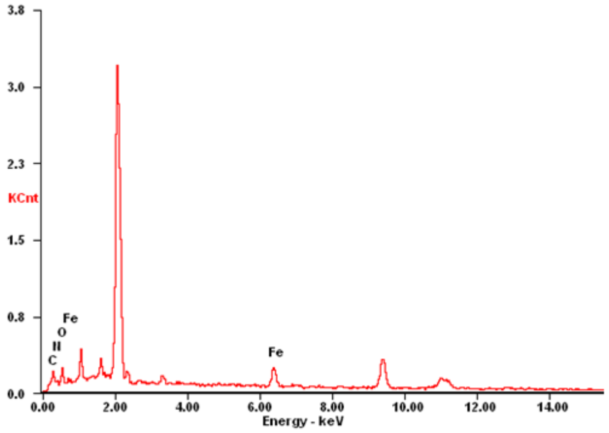

Figure S1. EDAX showing the elements content of the composite Fe_3O_4-Prussian blue.

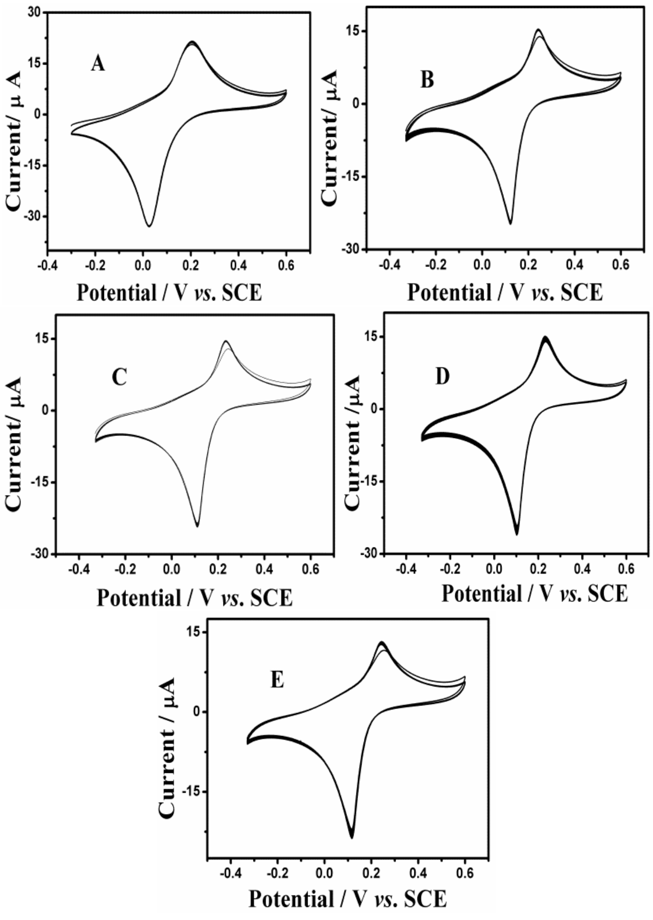

Figure S2. Multi-CVs of the Fe_3O_4-PB/GCE in 0.01 M Phosphate buffer solution (PBS) containing 0.1 M KCl at deferent pH values (**A**) 5.0, (**B**) 6.0, (**C**) 7.0, (**D**) 8.0, (**E**) 9.0.

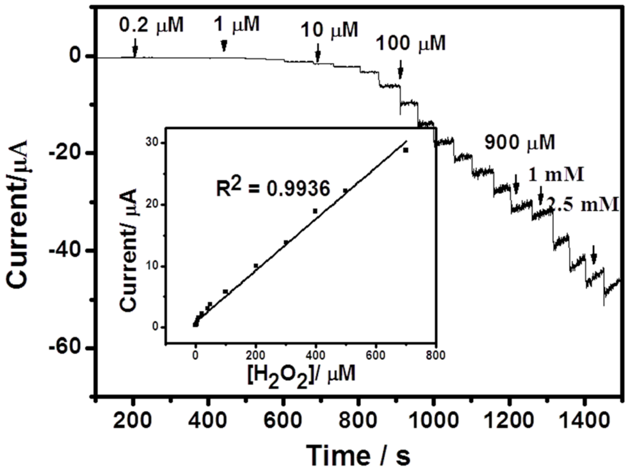

Figure S3. Current-time response of the Fe_3O_4-PB/GCE to the successive addition of H_2O_2 in 0.01 M PBS (pH 6.0) + 0.1 M KCl under stirring at −0.1 V. Insert: Plot of catalytic current *vs.* H_2O_2 concentration.

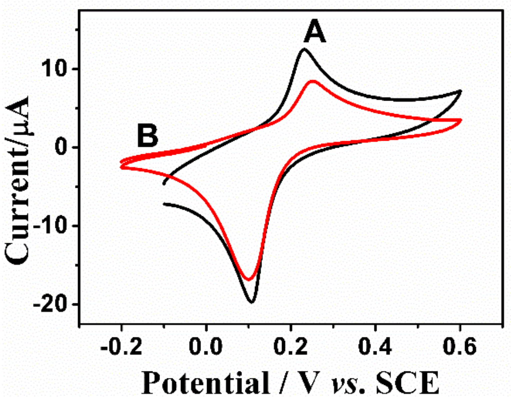

Figure S4. Typical CVs obtained at Fe_3O_4-PB/GCE (**A**) and GOD-BSA/Fe_3O_4-PB/GCE (**B**) in 0.01 M PBS (pH 6.0) + 0.1 M KCl. Scan rate: 50 mV·s^{-1}.

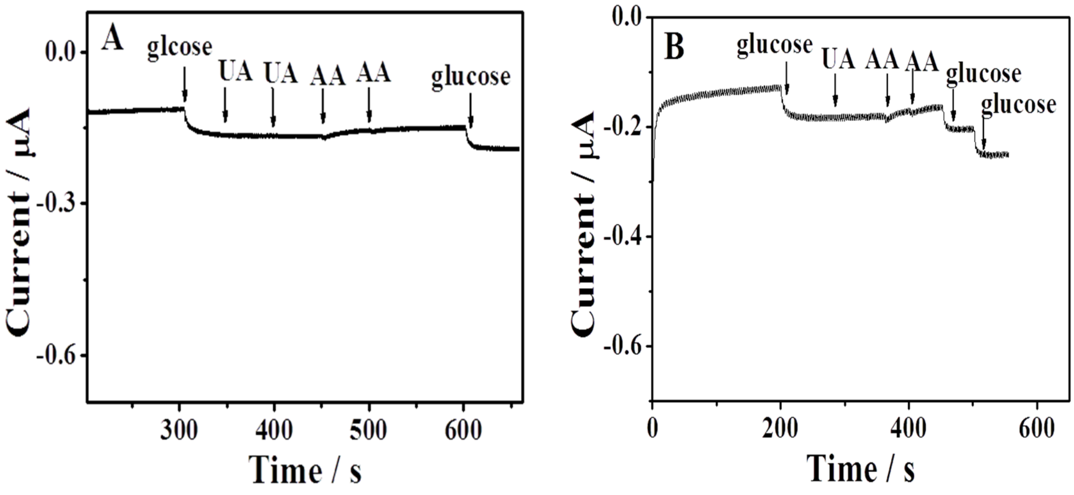

Figure S5. Typical Chronoaperometry (I-t) response of 0.025 μM glucose and (**A**) 0.1 μM AA and 0.1 μM UA (**B**) 0.2 μM AA and 0.2 μM UA at GOD-BSA/Fe_3O_4-PB/GCE. Applied Potential: −0.15 V.
